# Small cell ovarian carcinoma: genomic stability and responsiveness to therapeutics

**DOI:** 10.1186/1750-1172-8-33

**Published:** 2013-02-21

**Authors:** Lisa F Gamwell, Karen Gambaro, Maria Merziotis, Colleen Crane, Suzanna L Arcand, Valerie Bourada, Christopher Davis, Jeremy A Squire, David G Huntsman, Patricia N Tonin, Barbara C Vanderhyden

**Affiliations:** 1 Department of Cellular and Molecular Medicine, University of Ottawa, 451 Smyth Road, Ottawa, ON, K1H 8M5, Canada; 2 Centre for Cancer Therapeutics, Ottawa Hospital Research Institute, 501 Smyth Road, Ottawa, ON, K1H 8L6, Canada; 3 Department of Human Genetics, McGill University, 1205 Dr. Penfield Avenue, Montreal, QC, H3A 1B1, Canada; 4 The Research Institute of McGill University Health Centre, 2155 Guy Street, Montreal, QC, H3H 2R9, Canada; 5 Department of Medicine, McGill University, 687 Pine Avenue West, Montreal, QC, H3A 1A1, Canada; 6 Department of Pathology and Molecular Medicine, Queen’s University, 88 Stuart Street, Kingston, ON, K7L 3N6, Canada; 7 Department of Pathology and Laboratory Medicine, University of British Columbia, Vancouver, BC, Canada; 8 BC Cancer Agency, Vancouver, BC, Canada

**Keywords:** Small cell ovarian cancer, Cell line, Immunohistochemistry, BIN-67, Genomic anomalies

## Abstract

**Background:**

The biology of small cell ovarian carcinoma of the hypercalcemic type (SCCOHT), which is a rare and aggressive form of ovarian cancer, is poorly understood. Tumourigenicity, *in vitro* growth characteristics, genetic and genomic anomalies, and sensitivity to standard and novel chemotherapeutic treatments were investigated in the unique SCCOHT cell line, BIN-67, to provide further insight in the biology of this rare type of ovarian cancer.

**Method:**

The tumourigenic potential of BIN-67 cells was determined and the tumours formed in a xenograft model was compared to human SCCOHT. DNA sequencing, spectral karyotyping and high density SNP array analysis was performed. The sensitivity of the BIN-67 cells to standard chemotherapeutic agents and to vesicular stomatitis virus (VSV) and the JX-594 vaccinia virus was tested.

**Results:**

BIN-67 cells were capable of forming spheroids in hanging drop cultures. When xenografted into immunodeficient mice, BIN-67 cells developed into tumours that reflected the hypercalcemia and histology of human SCCOHT, notably intense expression of WT-1 and vimentin, and lack of expression of inhibin. Somatic mutations in *TP53* and the most common activating mutations in *KRAS* and *BRAF* were not found in BIN-67 cells by DNA sequencing. Spectral karyotyping revealed a largely normal diploid karyotype (in greater than 95% of cells) with a visibly shorter chromosome 20 contig. High density SNP array analysis also revealed few genomic anomalies in BIN-67 cells, which included loss of heterozygosity of an estimated 16.7 Mb interval on chromosome 20. SNP array analyses of four SCCOHT samples also indicated a low frequency of genomic anomalies in the majority of cases. Although resistant to platinum chemotherapeutic drugs, BIN-67 cell viability *in vitro* was reduced by >75% after infection with oncolytic viruses.

**Conclusions:**

These results show that SCCOHT differs from high-grade serous carcinomas by exhibiting few chromosomal anomalies and lacking *TP53* mutations. Although BIN-67 cells are resistant to standard chemotherapeutic agents, their sensitivity to oncolytic viruses suggests that their therapeutic use in SCCOHT should be considered.

## Background

Ovarian small cell carcinoma of the hypercalcemic type (SCCOHT) is a rare and highly aggressive form of ovarian cancer first reported in 1982 by Dickerson et al. [1]. The mean age of diagnosis is 23 years and the prognosis for these patients is generally poor, with a two year survival of less than 20% [2,3]. Although not considered a familial disease, there is a case report of an 11-year-old female diagnosed with SCCOHT, who had a strong family history of the disease, a reduction in the age of onset in the proband, and the absence of BRCA1/BRCA2 mutations [4]. While the incidence of SCCOHT is rare in the general population, it is the most common undifferentiated ovarian cancer in women under 40 years of age [5]. Its histogenesis is unknown, but the disease is associated with hypercalcemia in two-thirds of patients and the frequency of bilateral ovarian tumours is low [3]. Histologically, the tumours have a sheet-like arrangement of small, closely packed epithelial cells with 80% of cases containing variably sized follicle-like structures.

The rarity and aggressiveness of SCCOHT has lent itself poorly for study and therefore there are few reports on therapeutic strategies and no effective treatment regimens. While most patients undergo aggressive surgical resection followed by multi-agent, high dose chemotherapy, very few are cured [3]. Despite a rapid initial response to chemotherapy and radiation therapy, recurrence rates are high and those tumours tend to be less responsive to chemotherapy [3,5-7].

Although SCCOHT is morphologically similar to small cell carcinomas from other sites, its common expression of WT1 and lack of thyroid transcription factor (TTF)-1 allows it to be distinguished from other small cell cancers [8]. Immunohistochemical characterization of 15 SCCOHT showed frequent expression of p53, WT1 and epithelial markers, including epithelial membrane antigen, and less common to no expression of synaptophysin, S100 and inhibin [9]. The presence of p53 in 80-100% of SCCOHT suggests that *TP53* gene abnormalities may be involved in the genesis of this highly aggressive cancer [8,9], but mutational analysis has yet to be performed.

The cell line BIN-67, first reported in 1986 [10], was established from a metastatic pelvic nodule derived from a primary SCCOHT. In contrast to cell lines derived from ovarian serous adenocarcinomas, the BIN-67 cells express high levels of vimentin and respond to calcitonin with a >20-fold increase in cAMP [10]. BIN-67 appears to be the only SCCOHT cell line in existence and its further characterization could improve our understanding of this rare form of ovarian cancer. We have assayed the tumourigenic potential of BIN-67 cells and compared the tumours formed in a xenograft model to human SCCOHT. We also characterized their genomic content, performed a targeted gene mutation analysis, and tested their sensitivity to standard chemotherapeutic agents and to vesicular stomatitis virus (VSV) and the JX-594 vaccinia virus, both oncolytic viruses, which have been shown to be effective novel anti-cancer treatments in a variety of model systems [11-14].

## Methods

### Cell lines and SCCOHT samples

Primary mouse ovarian surface epithelial cells (MOSE) were isolated and cultured in α-MEM supplemented with 10% fetal calf serum (FCS; Sigma Chemical Co., St Louis, MO), epidermal growth factor and insulin-transferrin-selenium as described [15]. The platinum-sensitive human ovarian cancer cell line, A2780s, and its platinum-resistant derivative, A2780cp [16], were maintained in DMEM with 10% FCS. The BIN-67 cell line was obtained from Dr. S.R. Golding (Hospital for Special Surgery, New York) and cultured from frozen stock in DMEM supplemented with 20% FCS and enriched with 20% Ham’s F12 medium (Sigma Chemical Co.) as previously described [10].

Samples from four SCCOHT were obtained from the Children’s Oncology Group at Nationwide Children’s Hospital in Columbus, Ohio, The University Health Network (Dr. Blaise Clarke) and the Ovarian Cancer Research Program tissue bank in Vancouver, British Columbia, Canada. All resources provide access to samples through specific application to studies approved by institutional review boards.

### Spheroid formation assay

BIN-67 cells were tested for their ability to form spheroids by the hanging droplet method as previously described [17].

### Characterization of BIN-67 as a model of SCCOHT

BIN-67 cells (10^7^) in 1 mL of saline were injected intraperitoneally into 18 female 8-week-old Fox Chase SCID mice (CB-17 SCID, Charles Rivers Laboratories). When the mice reached a defined endpoint (large palpable mass), the tumours were removed, weighed and fixed in formalin. Histologic sections (5 μm) were prepared and either stained with hematoxylin and eosin to visualize morphology or immunostained for expression of cytokeratin (pre-diluted; Abcam, Cambridge, MA), vimentin (1:100; Dako, Carpinteria, CA), p53 (1:50; Santa Cruz Biotechnology; Santa Cruz, CA), KIT (CD117, 1:40; Dako), inhibin (1:25; Dako), WT1 (1:100; Dako), and the markers of neuroendocrine differentiation Pgp9.5 (1:3000; Millipore, Billerica, MA) and α-synaptophysin (1:400; BD Transduction, Mississauga, ON).

Blood samples were collected by saphenous vein puncture prior to BIN-67 xenograft and by cardiac puncture at necropsy and serum ionized calcium levels were measured using the i-STAT hand-held blood analyzer with EG7+ cartridges (Abbot Point of Care, Mississauga, Ontario, Canada) to determine if tumours derived from the BIN-67 cells cause hypercalcemia.

### High-density genotyping

Chromosomal anomalies in BIN-67 cells were inferred using the Infinium™ genotyping technology with the HumanHap300-Duo Genotyping BeadChip (Illumina, San Diego, CA, USA) as previously described [18]. This BeadChip contains about 318,000 genetic markers within approximately a 5 Kb median SNP spacing. Genotyping and imaging using BeadStudio Data Analysis software (Illumina, San Diego, CA, USA) were performed at the McGill University and Genome Quebec Innovation Centre (Montreal, QC). The February 2009 human reference sequence GRCh37/hg19 assembly (http://genome.ucsc.edu/cgi-bin/hgGateway) was used for the characterization of selected intervals. SNP array data for BIN-67 is available through the ArrayExpress Archive (http://www.ebi.ac.uk/arrayexpress/).

DNA from BIN-67 cells, four SCCOHT samples (T1, T2, T3 and T4), and a matched normal sample from one of the patients (T4-N) were genotyped using Affymetrix Genome-Wide Human SNP Array 6.0 and analyzed using CRMAv2 and HMMDosage as described previously [19,20]. To compare genomic anomalies across samples genotyped, the analyzed data was plotted and visualized using Circos [21].

### Karyotyping

Cytogenetic preparations from BIN-67 cultures were processed using standard methods and subjected to conventional G-banding and spectral karyotype (SKY) analysis [22]. Slides containing optimal metaphase preparations were aged for one week at room temperature and hybridized with the SKY-painting probes (Applied Spectral Imaging, Vista, Calif., USA) as per the manufacturer’s instructions. Image analysis and capture were performed using an AxioPlan Fluorescent Microscope and Spectral Karyotyping software (Applied Spectral Imaging).

### *TP53*, *KRAS* and *BRAF* mutation analyses

Mutation analysis of protein encoding regions and exon splice sites regions of *TP53*, and commonly mutated exons of *KRAS* and *BRAF* were sequenced and evaluated as described previously [18].

### Expression microarray analyses

Microarray expression analysis was performed using the GeneChip® Human Genome U133 Plus 2.0 Array (Affymetrix®, Santa Clara, CA) with total RNA from BIN-67 cells as described previously [18]. Hybridization and scanning were performed at the McGill University and Genome Quebec Innovation Centre (Montreal, QC). Gene expression levels were determined from the scanned images using Affymetrix® Microarray Suite (MAS) version 5.0 software expression algorithm (Affymetrix®) normalized as described previously [18]. Gene expression profiles were compared with Affymetrix U133 Plus 2.0 (MAS5.0) generated expression profiles (similarly normalized) from 10 human normal ovarian surface epithelial (OSE) cell brushings available in the ArrayExpress database (http://www.ebi.ac.uk/arrayexpress/), accession number E-GEOD-18520. Gene expression array data for BIN-67 is available through the ArrayExpress Archive (http://www.ebi.ac.uk/arrayexpress/).

### Treatment with chemotherapeutics and oncolytic viruses

To determine the effects of chemotherapeutic drugs on BIN-67 cells *in vitro*, MOSE, A2780cp, A2780s and BIN-67 cells were plated at a density of 1 × 10^4^ cells/100 μL in 96-well plates and after 24 hours the drugs were added (cisplatin: 0, 0.1, 1, 10, 100 μg/mL; carboplatin: 0. 1, 5, 10, 100, 200 μg/mL). The numbers of viable cells were determined 72 hours later using the CellTiter 96 AQ_ueious_ cell proliferation assay system.

To determine if oncolytic viruses could infect and kill the BIN-67 cells *in vitro*, the four cell lines were plated as described above. After 24 hours, the cells were washed twice with PBS and treated with an attenuated strain of VSV (VSV-Δ51; multiplicity of infections (MOI): 0, 0.001, 0.01, 0.1) and the vaccinia virus JX-594 (MOI: 0, 0.001, 0.01, 0.1) in serum-free media for 72 hours. The numbers of viable cells were determined using the metabolic indicator dye Alamar Blue (Serotec Ltd., Raleigh, NC), read with a Fluoroskan Ascent FL (Thermo Scientific, Rockland, IL). Both viruses were obtained from Dr. John Bell (Ottawa, ON) and were tagged with green fluorescent protein (GFP) to enable assessment of infection 48 hours after addition of virus [13,14].

### Statistical analysis

Experiments were performed at least three times in triplicate and statistical analyses (one-way ANOVA and Student’s t-tests) were performed using GraphPad Prism (version 3.02; GraphPad Software, San Diego, CA).

## Results

### BIN-67 cells are tumourigenic

In cell culture, BIN-67 cells are small with little cytoplasm. In hanging drop cultures, BIN 67 cells are capable of forming densely packed spheroids with an irregular but compact margin (Additional file 1: Figure S1), which is a feature exhibited by some epithelial ovarian cancer cell lines that are capable of forming tumours in mouse xenograft models [17].

**Figure 1:**
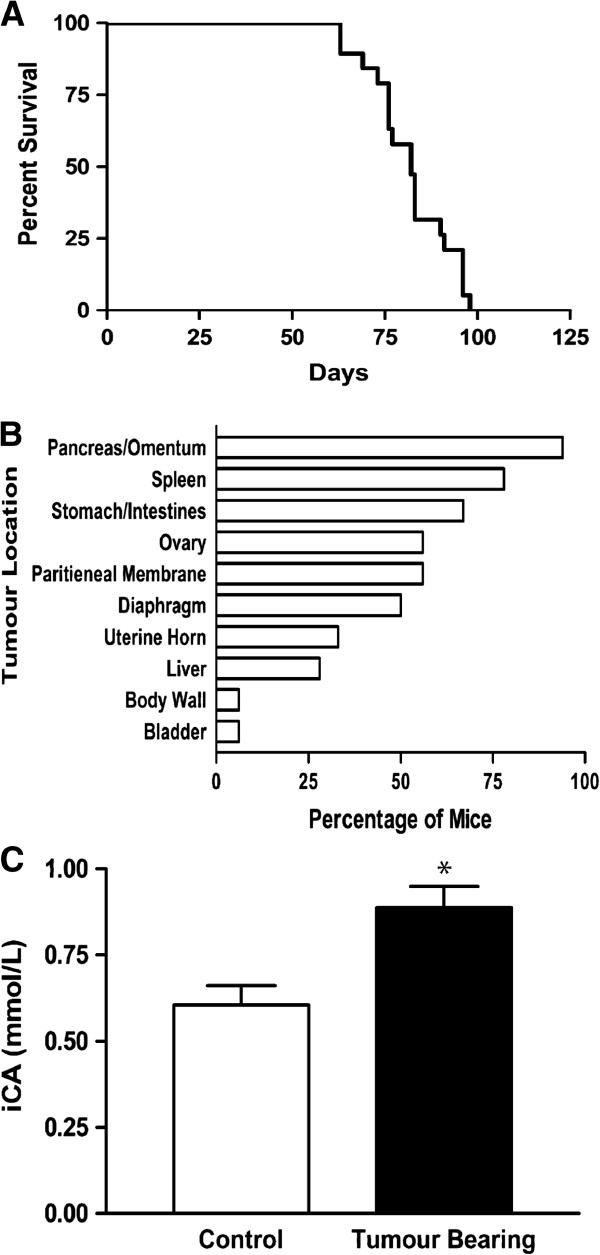
**Characterization of a BIN-67 xenograft model of SCCOHT. A)** Kaplan-Meier curve showing the survival of mice following intraperitoneal injection of BIN-67 cells. The cells form tumours with a median survival of 82 days. **B)** Quantification of tumour localization in the xenografts showed that the incidence of tumours is highest on the pancreas, omentum and spleen and is associated with the ovary in 56% of the mice. **C)** The formation of tumours is associated with a significant increase in serum ionized calcium (iCa) in the mice at endpoint (n=11) compared with levels measured in samples taken before the xenograft (n=4; * p<0.05).

**Figure 2:**
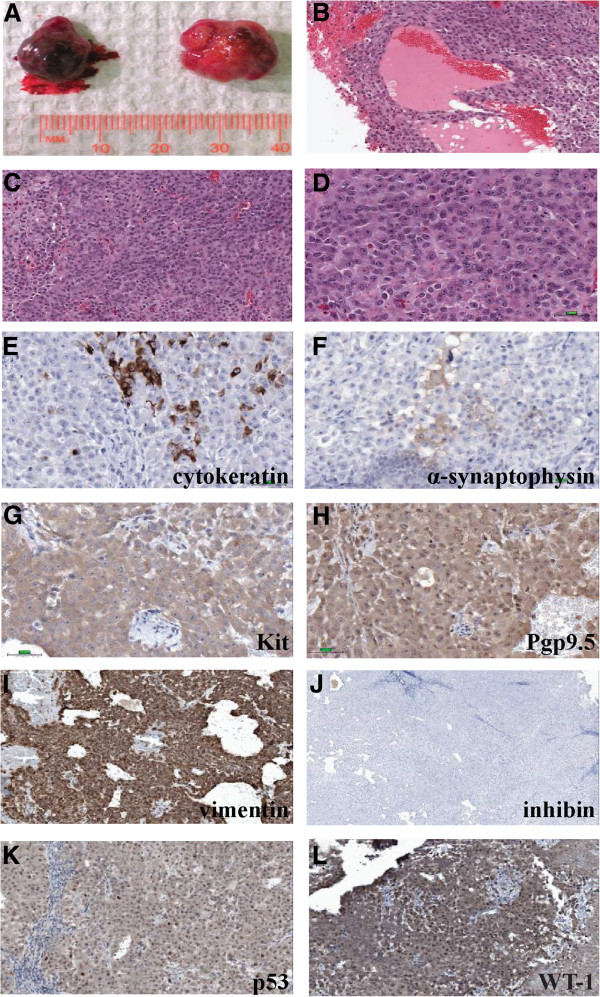
**Histology and expression of protein markers in BIN-67 tumours. A)** BIN-67 cells form large tumours in SCID mice. **B-D)** H&E staining of the tumours indicates that the histology resembles human small cell ovarian cancers, with homogeneous sheets of small cells and follicle-like structures **(B)**. **E**-**L**) Immunohistochemical staining for cytokeratin, α-synaptophysin, KIT, Pgp9.5, vimentin, inhibin, p53 and WT-1. **B**: 10X; **C**, **I**-**L**: 20X; **D**-**H**: 40X.

Intraperitoneal xenograft of the BIN-67 cells resulted in large palpable tumours in 18/18 mice. The median survival was 82 days (range: 61-96; Figure 1A) and the average tumour burden was 13% of body mass (Figure 2A). Tumours were associated with the surface of the diaphragm, spleen, pancreas, stomach, intestines, ovary and uterine horn with little to no ascites production (Figure 1B). The incidence of tumours was highest on the pancreas, omentum and spleen and was associated with the ovary in 56% of the mice. Similar to patients with SCCOHT, BIN-67 tumours in mice were associated with a significant increase in serum ionized calcium (iCa) at endpoint (n=11) compared with samples taken before the xenograft (n=4; p<0.05; Figure 1C).

SCCOHT is characterized by sheet-like arrangements of small, closely packed epithelial cells, wherein follicle-like structures can be found [2]. H&E staining of the BIN-67 tumours revealed follicle-like structures (Figure 2B) and small cells with scanty cytoplasm similar to the human disease (Figure 2C,D). Histological sections showed intense staining for vimentin and WT-1, moderate staining for KIT, Pgp9.5 and p53, and sporadic staining for cytokeratin and α-synaptophysin (Figure 2). In agreement with primary SCCOHT cancers, there is a lack of inhibin staining which helps to distinguish this tumour type from granulosa cell tumours that are typically inhibin immunoreactive. These observations are consistent with low expression values for *KIT*, *UCHL1* (Pgp9.5), *TP53*, and *INHA* (inhibin α) in contrast to higher levels of expression of *VIM* and *WT1* as determined using gene expression microarray analyses (Additional file 2: Figure S2).

### BIN-67 and SCCOHT exhibit low level chromosomal anomalies

**Figure 3:**
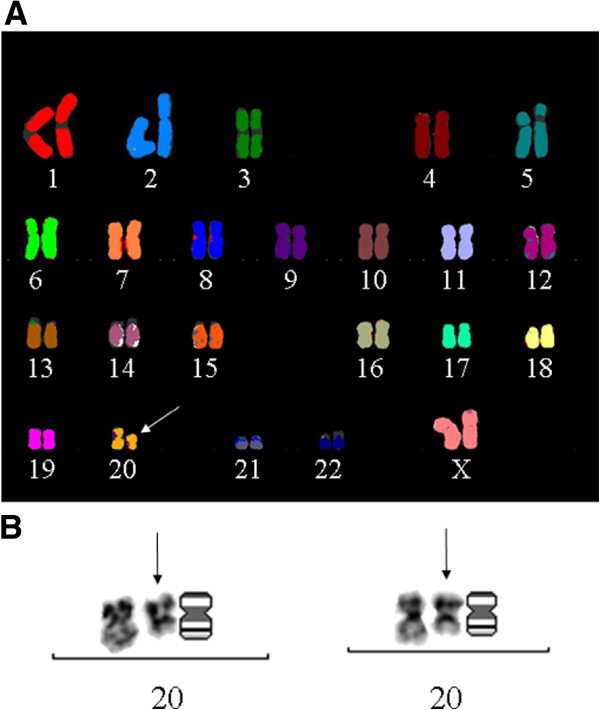
**Spectral karyotype analysis of BIN-67 cells.** Spectral karyotype analysis of BIN-67 cells exhibiting normal diploid population with the exception of a shorter chromosome 20 contig shown with an arrow **(A)** and Giemsa stained image of chromosome 20 contigs, which also show an abnormally shortened contig as shown with an arrow **(B)**.

SKY analysis of BIN-67 cells revealed a predominantly diploid (46,XX) cell population (>95% of cells), and a sub-population of tetraploid (92, XXXX) cells. The cells show a normal karyotype with the exception of a visibly shorter chromosome 20 contig, which was evident from both SKY analysis and Giemsa staining (Figure 3).

**Figure 4:**
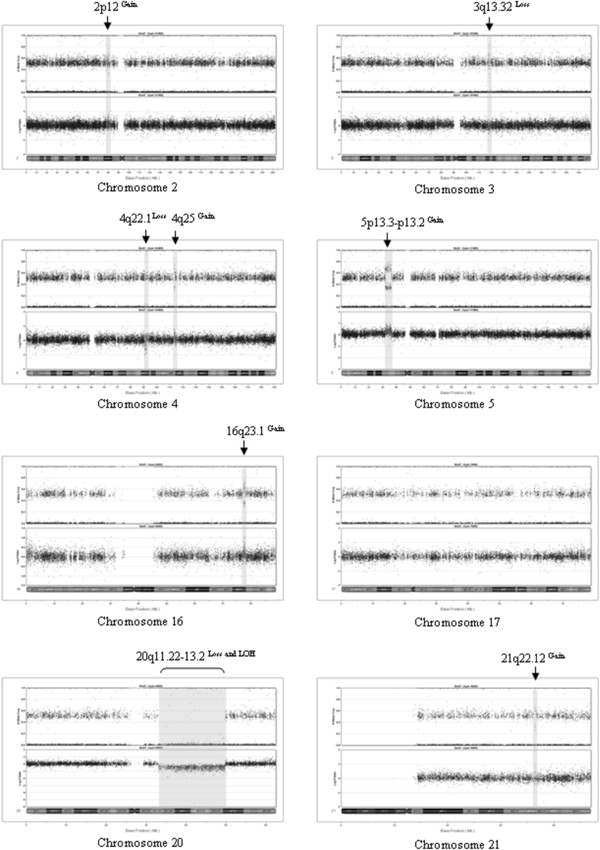
**Illumina BeadChip array analysis of BIN-67.** The results for each marker (SNP) are shown aligned to its genomic position along a chromosome (Mb). The top image for each panel contains the B allele frequency (zygosity) and bottom image the Log R ratio (copy number) for each marker analyzed. Data is absent for centromeric regions as these regions are not represented by SNP arrays. Gains or losses (gray shaded regions) were observed for all chromosomes indicated with the exception of chromosome 17.

High-density SNP array analyses based on the Infinium™ HumanHap300-Duo BeadChip was used to further characterize genomic anomalies in BIN-67 cells (Figure 4). As summarized in Table 1, nine discrete copy number variations were detected ranging in size from approximately 97 Kb to 16.8 Mb. Copy number gain involved 2p12, 4q25, 5p13.3-p13.2, 16q23.1, and 21q22.12, and copy number loss involved 3q13.32, 4q22.1, and 20q11.22-q13.2. Loss of heterozygosity was detectable with the extensive region of copy number loss overlapping 20q11.22-q13.2. This observation along with karyotype analysis (Figure 3) suggests that this chromosome had undergone an intrachromosomal deletion.

**Table 1:** Catalogue of copy number variations and LOH in BIN-67 cell line

**Chr**	**SNP start; SNP end**	**# of SNPs**	**Region (Mb)**	**Size (Kb)**	**LOH**	**CNV**	**# RefSeq**	**RefSeq genes**	**Gene expression ratio BIN-67/OSE >2**
2p12	rs12713994; rs6729700	31	80.1-80.2	97.1	-	Gain	1	CTNN2A*	none
3q13.32	rs9810432; rs2868975	19	116.8-116.9	107.3	-	Loss	0		n/a
4q22.1	rs2583985; rs1838227	30	90.7-91.0	267.3	-	Loss	2	SNCA*, MMRN1	n/a
4q22.1	rs10017262; rs6849036	20	91.8-91.9	116.1	-	Loss	1	FAM190A *	n/a
4q25	rs10516583; rs895763	69	113.0-113,8	75.7	-	Gain	8	C4orf32, AP1AR, TIFA, ALPK1, NEUROG2, C4orf21, LARP7, ANK2*	ALPK1, NEUROG2, LARP7
5p13.3-p13.2	rs6450864; rs10454863	545	31.8-36,2	4450.9	-	Gain	28	PDZD2, GOLPH3, MTMR12, ZRF, SUB1, NPR3, LOC340113, TARS, ADAMTS12, RXFP3, SLC45A2, AMACR, C1QTNF3, RAI14, TTC23L, RAD1, BRIX1, DNAJC21, AGXT2, PRLR, SPEF2, IL7R, CAPSL, UGT3A1, UGT3A2, LMBRD2, SKP2, NADKD1*	PDZD2, GOLPH3, ZRF, SUB1, PRLR, SKP2
16q23.1	rs6564596; rs4888943	133	78.8-79.3	462.7	-	Gain	1	WWOX*	none
20q11.22-q13.2	rs1998233; rs6021435	1 886	33.6-50.4	16 742.9	+	Loss	187	TRPC4AP*, EDEM2, PROCR, MMP24, EIF6, FAM83C, UQCC, GDF5, CEP250, C20orf173, ERGIC3, FER1L4, SPAG4, CPNE1, RBM12, NFS1, ROMO1, RBM39, PHF20, SCAND1, C20orf152, LOC647979, EPB41L1, C20orf4, DLGAP4, MYL9, TGIF2, TGIF2-C20ORF24, C20orf24, SLA2, NDRG3, DSN1, C20orf117, C20orf118, SAMHD1, RBL1, C20orf132, RPN2, GHRH, MANBAL, SRC, BLCAP, NNAT, LOC100505783, CTNNBL1, VSTM2L, TTI1, RPRD1B, TGM2, KIAA1755, BPI, LBP, LOC388796, SNHG11, RALGAPB, ADIG, ARHGAP40, SLC32A1, ACTR5, PPP1R16B, FAM83D, DHX35, LOC339568, MAFB, TOP1, PLCG1, ZHX3, LPIN3, EMILIN3, CHD6, PTPRT, SRSF6, L3MBTL1, SGK2, IFT52, MYBL2, GTSF1L, TOX2, JPH2, C20orf111, LOC100505783, GDAP1L1, FITM2, R3HDML, HNF4A, TTPAL, SERINC3, PKIG, ADA, LOC79015, WISP2, KCNK15, RIMS4, YWHAB, PABPC1L, TOMM34, STK4, KCNS1, WFDC5, WFDC12, PI3, SEMG1, SEMG2, SLPI, MATN4, RBPJL, SDC4, SYS1, SYS1-DBNDD2, TP53TG5, DBNDD2, PIGT, WFDC2, SPINT3, WFDC6, SPINLW1-WFDC6, SPINLW1, WFDC8, WFDC9, WFDC10A, WFDC11, WFDC10B, WFDC13, SPINT4, WFDC3, DNTTIP1, UBE2C, TNNC2, SNX21, ACOT8, ZSWIM3, ZSWIM1, C20orf165, NEURL2, CTSA, PLTP, PCIF1, ZNF335, MMP9, SLC12A5, NCOA5, CD40, CDH22, SLC35C2, ELMO2, MKRN7P, LOC100240726, ZNF334, C20orf123, SLC13A3, TP53RK, SLC2A10, EYA2, , ZMYND8, LOC100131496, NCOA3, SULF2, LOC284749, PREX1, ARFGEF2, CSE1L, STAU1, DDX27, ZNFX1, NCRNA00275, , KCNB1, PTGIS, B4GALT5, SLC9A8, SPATA2, RNF114, SNAI1, TMEM189-UBE2V1, UBE2V1, TMEM189, CEBPB, LOC284751, PTPN1, FAM65C, PARD6B, BCAS4, ADNP, DPM1, MOCS3, KCNG1, NFATC2, ATP9A	n/a
									
21q22.12	rs2212922 ; rs7280062	20	37.5-37.6	108.3	-	Gain	1	LOC100133286, LOC100506428, CBR3, DOPEY2*	none

LOH: Loss of heterozygosity; CNV: Copy number variation; n/a: not applicable; * Refseq genes in breakpoints; The Human February 2009 [hg19] Assembly of the UCSC genome browser, http://genome.ucsc.edu/ was used.

**Figure 5:**
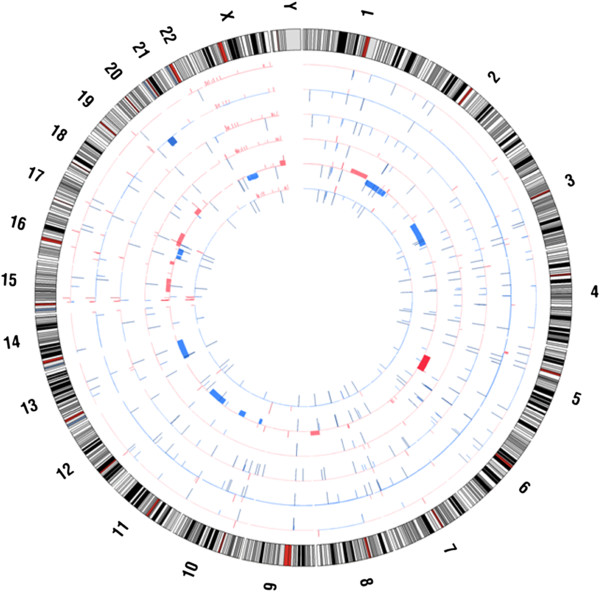
**Circos plot of Affymetrix SNP array analysis performed on the BIN-67 cells and four SCCOHT samples.** The track order (outside in) is patient tumour sample T2, BIN-67, patient tumour samples T1, T3, T4 and the matched normal sample, T4-N.

To compare genomic landscapes, Affymetrix SNP 6.0 array analysis was performed on the BIN-67 cells and four SCCOHT samples: T1, T2, T3 and T4, and one matched normal sample (T4-N). A summary of the copy number variations is shown aligned to chromosomal position, displayed in a Circos plot in Figure 5. Discrete copy number variations were observed with all samples. Sample T4 was notable for exhibiting the largest number of genomic variations. Notable is that SNP array results of BIN-67 cells were concordant with that derived using the Infinium platform. Though the large 20q11.22-q13.2 deletion observed in BIN-67 was not detectable in any of the tumour specimens, there were many discrete anomalies (both gains and losses) that overlapped similar regions in the tumour samples and the BIN-67 sample that were not observed in the reference normal sample, suggesting that they may be unique to the development of SCCOHT. In total the BIN-67 sample had 100 discrete gains or losses, with 90 of these not found in the normal sample. Of these 90 gains or losses, 34 were found in at least one of the tumour samples, and one loss (chromosome 1; 151028548-151035325) was shared by all 4 tumour samples but not with the normal sample. Examples of some shared copy number variations are summarized in Additional file 3: Table S1 and the complete Affymetrix SNP 6.0 array CRMAv2 and HMMDosage analysis can be found in Additional file 4: Table S2.

### BIN-67 mutational spectra exclude conventional genes

A sequence analysis was performed to determine if BIN-67 harboured mutations in *TP53*, *KRAS* and *BRAF* genes previously shown mutated in epithelial ovarian carcinomas [18]. Mutation analysis did not detect any variants in the protein coding regions of *TP53* nor in commonly mutated exons of *KRAS* and *BRAF.*

### Gene expression profile targeted genomic regions affected in BIN-67

Transcriptome analysis of BIN-67 proved to be a challenge as there is no corresponding normal tissue available for comparison. We therefore focused our analysis on investigating the expression profile of genes located within regions exhibiting copy number gains, as these regions may contain genes exhibiting increased expression due to alterations in copy number as demonstrated in our previous analyses of ovarian cancer cell lines exhibiting distinct genomic amplification events [23], (Table 1). Although the cell type of origin of SCCOHT is not known, we compared the expression profiles to publicly available data representing OSE samples derived using the same gene expression microarray platform. Only genes mapping to the 4q25 and 5p13.3-p13.2 exhibited evidence of expression greater than 2-fold when compared with OSE samples (Additional file 5: Figure S3). Of the three genes *ALPK1*, *NEUROG2*, and *LARP7* exhibiting higher levels of expression in the 4q25 region, only *ALPK1* and *NEUROG2* consistently exhibited greater than 2-fold levels of expression when compared with each OSE sample. Of 5 of 28 genes that map to the 5p13.3-p13.2 region and exhibit greater than 2-fold difference in gene expression, only *PDZD2*, *SUB1*, *PRLR* and *SKP2* consistently exhibited expression greater than 2-fold in two-way comparisons to each OSE sample (Additional file 5: Figure S3).

### Treatment with standard chemotherapeutics and oncolytic viruses

**Figure 6:**
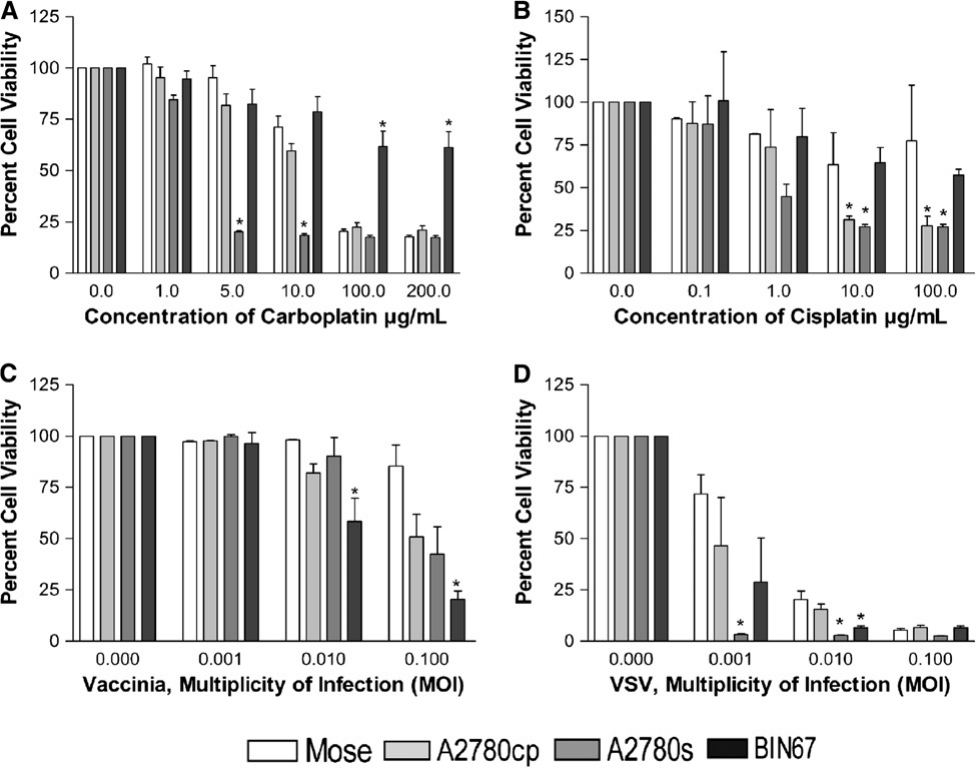
**Cell viability of MOSE, A2780s, A2780cp and BIN-67 cells after treatment with chemotherapeutics and oncolytic viruses.** Cell viability of MOSE, A2780s, A2780cp and BIN-67 cells after treatment for 72 hours with carboplatin **(A)**, cisplatin **(B)**, the vaccinia virus JX-594 **(C)** and VSV **(D)** at the concentrations/MOIs indicated. Viability was assessed using MTS assays, and bars show mean ± SEM of viable cells with data normalized to 100% for untreated cells. Bars indicated with an asterisk identify treatment groups that are significantly different from MOSE cells at that same concentration/MOI (p<0.05).

The high mortality of women with SCCOHT indicates a strong need to improve the current strategies for treatment. To investigate the response of BIN-67 cells to standard and novel treatments, we first examined the effect of carboplatin and cisplatin on BIN-67 viability. As controls, chemosensitive A2780s cells and chemoresistant A2780cp cells confirmed their differential sensitivity to carboplatin at concentrations of 5 and 10 μg/mL, with less than 25% viability for both cell lines at higher concentrations (Figure 6A). The normal MOSE cells remained viable (>70%) after exposure to concentrations up to 10 μg/mL, but viability was reduced at higher concentrations. In contrast, BIN-67 cells were exceptionally resistant, with 61% viability at the highest concentration of carboplatin tested (Figure 6A) and similar resistance to cisplatin-induced cell killing (Figure 6B). BIN-67 cells proved to be resistant to a non-platinum drug as well, with cell viability reduced to 64% after 72 hours of exposure to 10 μM taxol, compared with only 22% of A2780cp cells remaining viable (data not shown).

Since BIN-67 cells were resistant to conventional chemotherapeutics, we tested their response to novel treatments. Two oncolytic viruses, the vaccinia virus JX-594 and VSV, were tested for cytotoxic effects on the four cell lines. Treatment with GFP-tagged viruses showed that BIN-67 cells could be readily infected with both of these viruses (Additional file 6: Figure S4). Infection with JX-594 significantly reduced BIN-67 cell viability at an MOI of 0.01, and this viability was reduced further to just 20% when the cells were exposed to an MOI of 0.1. The sensitivity of BIN-67 to JX-954 was greater than the response of the A2780s and A2780cp cells, whereas normal MOSE remained unaffected (Figure 6C). BIN-67 cells were also very sensitive to VSV-induced cell killing, with a significant (75%) decrease in viability evident at an MOI of 0.001, and just 7% cell viability at the higher MOIs (Figure 6D).

## Discussion

Small cell carcinoma is a rare tumour that is usually associated with the lung and/or cervix in females, but can occur rarely in the ovary. The biology of SCCOHT is poorly understood, but the relatively young age of SCCOHT patients and the difficulties associated with treating them warrant investigation of this very aggressive type of cancer. Given the challenges of studying the rare forms of cancer in humans, we have established and characterized a unique xenograft model of SCCOHT. Validation of this model was achieved by demonstrating its similarity to the human disease in its histological and immunohistochemical features, as well as the exhibition of hypercalcemia, which occurs in the majority of SCCOHT patients. The ability of BIN-67 cells to form spheroids in hanging drop cultures also has been observed in epithelial ovarian cancer cell lines that are tumourigenic in mouse xenograft models [17]. Though the factors involved are not known, comparative transcriptome analyses of epithelial ovarian cancer cell line models have shown that spheroids and tumour xenografts were more similar in their expression profiles than when compared with transcriptomes derived from cell lines grown as monolayers in cultures [17,24]. Notable also is that suppression of tumourigenic potential in at least one ovarian cancer cell line resulted in loss of both spheroid forming capacity and ability to form mouse tumour xenografts [24]. The growth phenotypes exhibited by BIN-67 will enable further study of this unique model of SCCOHT to address progression and treatment of this disease.

Immunohistochemical staining of the BIN-67-derived tumours revealed a diagnostic expression pattern that is similar to that reported in humans, notably intense expression of WT-1 and vimentin and lack of expression of inhibin. The moderate levels of staining for p53 and KIT also resemble human cancers [8,9]. In agreement with the 90% of human SCCOHT tumours that are immunoreactive for cytokeratins [5], we observed cytokeratin staining in the BIN-67 tumours, when detected using pan-cytokeratin antibodies. The limited, sporadic staining for α-synaptophysin was also as expected, since this is a neuroendocrine marker and is not often found in human SCCOHT [9].

Small cell carcinomas of the ovary are distinguished into two types: hypercalcemic and pulmonary type (SCCOPT). SCCOPT are so designated because of their similarities to small cell carcinomas of the lung [2]. SCCOHT differ markedly from SCCOPT and from small cell lung cancers in clinical presentation, histological features and immunohistochemical markers. Small cell carcinomas of the lung and cervix are often associated with neuroendocrine differentiation as manifested by their histologic growth pattern, ultrastructure and expression of neuroendocrine markers, whereas the current consensus is that SCCOHT are not neuroendocrine in type [2]. The moderate staining for PGP9.5, a neuroendocrine marker, in the BIN-67 tumours was therefore unexpected. PGP9.5 (*UCHL-1*) is a neurospecific peptide that functions to remove ubiquitin from ubiquitinated proteins and prevents them from targeted degradation by proteasomes. It is abundantly expressed in small cell lung and cervical cancers [25,26], which are both neuroendocrine tumours. To our knowledge, the expression of PGP9.5 has not previously been examined in SCCOHT, and so it remains unclear whether its expression in the BIN-67 tumours is an unanticipated feature of this type of tumour or whether xenografting these cells had modified their behaviour.

Known as a paraneoplastic disorder, humoral hypercalcemia presents in a variety of cancers, including squamous cell carcinoma of lung, adenocarcinoma of gastrointestinal tract, and small cell carcinoma of ovary. The hypercalcemia may be caused by the secretion of parathyroid hormone related protein (PTHrp) by the tumour cells, which can act through PTH receptors to mediate the calcium release [27-30]. The hypercalcemia observed in the mice with BIN-67-derived tumours therefore reflects well the hypercalcemia that occurs in the majority of patients with SCCOHT.

BIN-67 lacks the mutational spectrum characteristic of the major histopathological subtypes of ovarian cancer. The low level of chromosomal anomalies and absence of *TP53* mutations distinguishes BIN-67 cells from high-grade ovarian serous carcinomas [31,32]. The absence of *KRAS/BRAF* mutation also distinguishes BIN-67 from low-grade ovarian serous carcinomas and mucinous cancers [33,34]. The low level of chromosomal anomalies was also observed with three of the four SCCOHT, suggesting that a modest alteration in genomic landscape may be characteristic of this type of cancer. One genomic anomaly was common to all four SCCOHT patient tumour samples and the BIN-67 cells, but not the matched normal sample. This loss occurred in the region of chromosome 1 containing the *MLLT11* gene (myeloid/lymphoid or mixed-lineage leukemia (trithorax homolog, *Drosophila*); translocated to, 11), making this a potentially interesting gene to study in SCCOHT. *MLLT11*, also known as *AF1Q*, has been reported to be an oncogenic factor involved in the development of leukemia and thyroid tumours, and breast cancer metastasis [35-37].

The 5p13.3-p13.2 interval gained in BIN-67 has been shown to be amplified in various cancer types, including ovarian cancer [31]. Although various amplicons have been described, *PDZD2*, *GOLPH3*, *PRLR* and *SKP2* have emerged as potential targets, which is interesting given their gene expression profile in BIN-67 cells. PDZD2 may be involved in intracellular signaling and is overexpressed in prostate cancer and associated with the initiation or early events in tumourigenesis [38]. Paradoxically, it has recently been shown to induce either senescence or apoptosis in cancer cells via transcriptional activation of *TP53*[39]. *GOLPH3*, which encodes a peripheral membrane protein of the Golgi stack and may have a regulatory role in Golgi trafficking, was recently shown to enhance growth-factor-induced mTOR signaling and consequently alter response to rapamycin, an mTOR inhibitor, in cancer cells [40]. Further investigation of BIN-67 cells for sensitivity to rapamycin is warranted given the potential of identifying new targets for chemotherapy as this drug is in clinical use.

*PRLR* encodes the prolactin receptor, which may function to modulate endocrine and autocrine effects of prolactin in normal and cancer tissues, and has been extensively studied as a potential therapeutic target in breast cancer [reviewed in [41]. It was recently shown to be associated with increasing survival and migration of ovarian cancer cells and was proposed as a potential therapeutic target for receptor antagonists for ovarian cancer [42]. Although *PDZD2* and *GOLPHA3* are potential targets of amplification in BIN-67, *SKP2,* which encodes a member of the F-Box protein family S-phase kinase-associated protein 2 (p45), is an established oncogene, and has been extensively studied as a therapeutic target [43]. SKP2 protein overexpression in epithelial ovarian cancers has been reported [44,45] and this expression signature has been proposed as a prognostic factor [46,47].

The gain of the 4q25 interval in BIN-67 is interesting, as it contains *LARP7,* which encodes PIP7S, recently shown to bind and stabilize all the nuclear 7SK RNA leading to inactivation of a general transcription factor P-TEFb that stimulates RNA polymerase II elongation and cotranscriptional processing of pre-mRNA [48]. Knockdown of PIP7S with shRNA in a normal human mammary epithelial cell line shifts the P-TEFb equilibrium and causes disrupted epithelial differentiation, P-TEFb-dependent malignant transformation and activation of key tumour-related genes, which is consistent with the tumour suppressor function of its *Drosophila* homolog [49].

The prognosis of women with SCCOHT is very poor, largely due to the lack of effective treatments; however, there have been some case reports of long-term survival with a multi-modality approach to treatment. Tewari et al. [50] reported a case of SCCOHT diagnosed during pregnancy that was treated with cytoreductive surgery and multi-agent chemotherapy (vinblastine, cisplatin, cyclophosphamide, bleomycin, doxorubicin, and etoposide). The patient was alive and without evidence of disease 5.5 years after diagnosis. Treatment with conservative surgery and the same chemotherapy agents resulted in a 19 year old patient with advanced-stage (Stage IIIC) SCCOHT doing well more than 2 years after completion of treatment [51]. A more recent multi-national retrospective analysis of the management of 17 SCCOHT patients resulted in the recommendation of multi-modality treatment approaches including surgery and chemotherapy with the addition of radiotherapy either sequentially or concurrently [52].

Despite these reports, the prognosis and outcome for the majority of patients diagnosed with SCCOHT remains poor and better treatment options are needed. Oncolytic virus therapy is an innovative alternative to conventional cancer therapies and is based on the concept that it is possible to select or engineer viruses to preferentially replicate in and kill tumour cells [11,53-55]. This cancer cell selectivity is at least in part due to an acquired, tumour-specific defect in cellular innate antiviral responses [56]. Oncolytic vaccinia viruses are currently positioned for testing in Phase III clinical trials [13] and VSV is a prototypical rhabdovirus that grows poorly in normal tissues but replicates efficiently in cells lacking an intact IFN response [57]. Both oncolytic viruses efficiently infected the BIN-67 cells, resulting in a loss of cell viability by as much as 93%. In contrast, BIN-67 cells showed marked resistance to carboplatin, cisplatin and taxol, chemotherapeutic agents commonly used for the treatment of ovarian cancer. This resistance was greater than that shown by the A2780cp cells, an epithelial ovarian cancer cell line commonly used to study the mechanisms of chemoresistance. These results suggest that platinum drugs may not be the optimal treatment for SCCOHT and that therapeutic use of oncolytic viruses should be investigated further for the treatment of SCCOHT, as well as other ovarian cancers.

## Conclusions

Although the establishment of other SCCOHT-derived cell lines has been periodically reported [58,59], it appears that none have been investigated beyond the initial reports. The demonstration that BIN-67 cells can form spheroids *in vitro* and tumours *in vivo* provide two novel model systems with potential value for preclinical drug screening. Spheroid models have been shown to better mimic the *in vivo* tumour gene expression patterns than monolayer cultures, as have been demonstrated in comparative transcriptome profiling analyses [17,24]. BIN-67 cells therefore provide the rare opportunity to investigate the biology of SCCOHT cells and tumours. Further characterization of this rare form of ovarian cancer may help to determine the origins of this disease as well as increase the number of therapeutic options.

## Competing interests

The authors declare that they have no competing interests.

## Authors’ contributions

LFG carried out the xenografts, measured iCa levels and prepared the initial draft of the manuscript. KG performed the spheroid formation assays, analysed the gene expression microarray and Illumina SNP array, drafted sections of manuscript and edited drafts of the manuscript. MM and CD performed and analysed the experiments testing platinum chemotherapies and oncolytic viruses. CC performed all the immunohistochemistry. VB performed the initial experiments testing VSV on cell lines. SLA carried out the gene specific mutation analyses and Illumina SNP array analyses. JAS analyzed the spectral and Giemsa karyotyping. DGH performed and analysed the Affymetrix SNP array. PNT analysed the gene expression microarray and Illumina SNP array, drafted sections of manuscript, and edited drafts of the manuscript. BCV conceived of the conceptual plan for the project and experimental design for the xenografts and therapeutic testing; wrote sections and edited drafts of the manuscript. All authors read and approved the final manuscript.

## Supplementary Material

Additional file 1: Figure S1. Morphological appearance of spheroids in hanging droplets cell culture assays four or five days after seeding BIN-67 cells in growth medium (20x magnification). (TIFF 446 kb) Click here for file

Additional file 2: Figure S2. Affymetrix microarray gene expression values of selected probe sets representing genes examined by immunohistochemistry analysis in tumour xenografts of BIN-67 cells. (TIFF 12 kb) Click here for file

Additional file 3: Table S1. Examples of SNPs shared between BIN-67 and at least 1 tumour sample, but not the matched normal sample identified by CRMAv2 and HMMDosage analysis of the Affymetrix Genome-Wide Human SNP Array 6.0. (PDF 23 kb) Click here for file

Additional file 4: Table S2. Complete Affymetrix Genome-Wide Human SNP Array 6.0 CRMAv2 and HMMDosage analysis results. (XLSX 78 kb) Click here for file

Additional file 5: Figure S3. Affymetrix microarray gene expression values of probe sets representing genes exhibiting at least a two-fold increased expression in BIN-67 cells relative to mean of 10 OSE samples. The differentially expressed genes map within the 4q24 and 5p13.3-p13.2 intervals exhibiting increased copy number as inferred by Illumina BeadArray genotyping analysis. (TIFF 14 kb) Click here for file

Additional file 6: Figure S4. BIN-67 cell infection by VSV (**Figure S4A**) and vaccinia virus JX-594 (**Figure S4B**)**.** BIN-67 cells were compared with A2780s and A2780cp ovarian cancer cell lines and normal MOSE for their susceptibility to infection by GFP-tagged virus. After 48 hours of infection, BIN-67 cells were found to express GFP after exposure to all MOI, indicating that these cells were as readily infected with VSV as the two other cancer cell lines. At this time, the cells already display a rounded and detached morphology, suggestive of cells undergoing cell death. Normal MOSE cells were more resistant to infection by VSV and JX-594. (TIFF 822 kb) Click here for file
