# Supplementary figures and images for: Small cell ovarian carcinoma: genomic stability and responsiveness to therapeutics
Source: Orphanet J Rare Dis. 2013 Feb 21;8:33. doi: 10.1186/1750-1172-8-33 (PMC3635907; doi:10.1186/1750-1172-8-33)

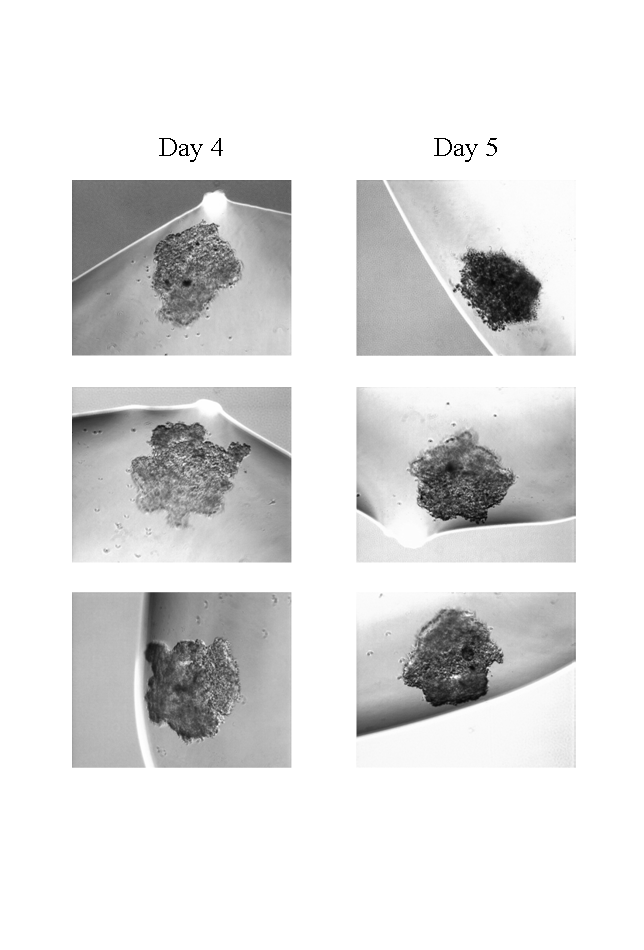

Supplement: Additional file 1: Figure S1. — Morphological appearance of spheroids in hanging droplets cell culture assays four or five days after seeding BIN-67 cells in growth medium (20x magnification). (TIFF 446 kb) [file 1750-1172-8-33-S1.tiff]

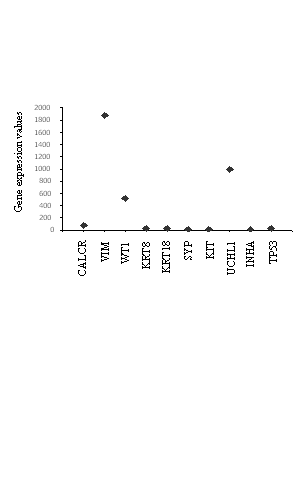

Supplement: Additional file 2: Figure S2. — Affymetrix microarray gene expression values of selected probe sets representing genes examined by immunohistochemistry analysis in tumour xenografts of BIN-67 cells. (TIFF 12 kb) [file 1750-1172-8-33-S2.tiff]

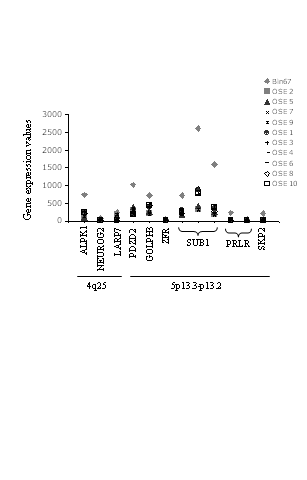

Supplement: Additional file 5: Figure S3. — Affymetrix microarray gene expression values of probe sets representing genes exhibiting at least a two-fold increased expression in BIN-67 cells relative to mean of 10 OSE samples. The differentially expressed genes map within the 4q24 and 5p13.3-p13.2 intervals exhibiting increased copy number as inferred by Illumina BeadArray genotyping analysis. (TIFF 14 kb) [file 1750-1172-8-33-S5.tiff]

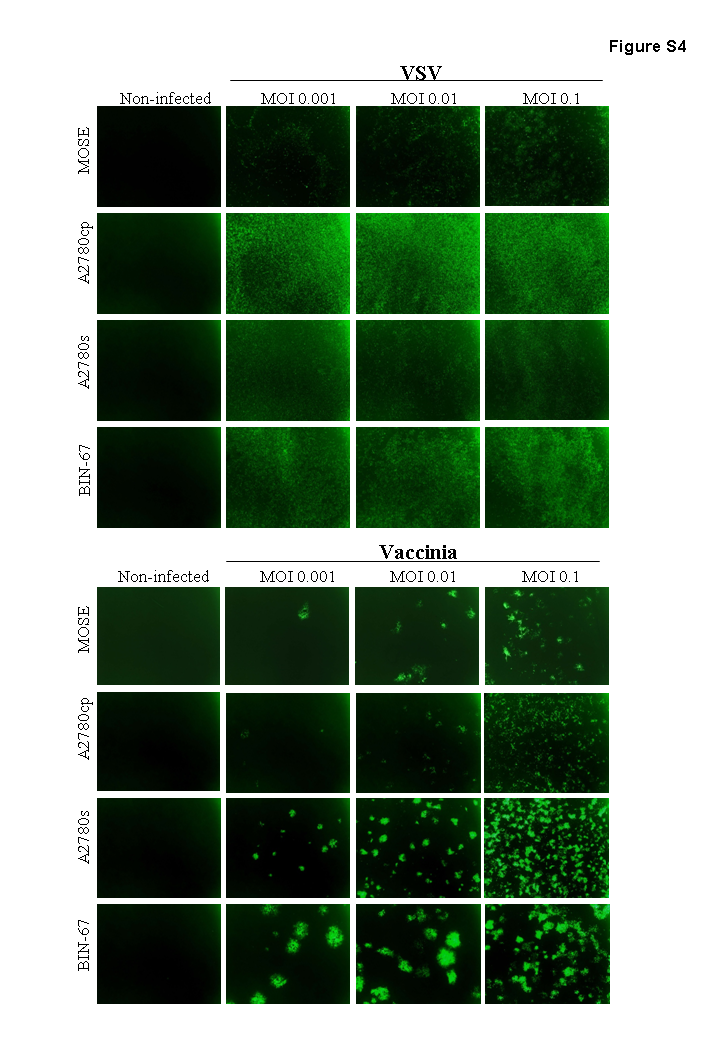

Supplement: Additional file 6: Figure S4. — BIN-67 cell infection by VSV (Figure S4A) and vaccinia virus JX-594 (Figure S4B). BIN-67 cells were compared with A2780s and A2780cp ovarian cancer cell lines and normal MOSE for their susceptibility to infection by GFP-tagged virus. After 48 hours of infection, BIN-67 cells were found to express GFP after exposure to all MOI, indicating that these cells were as readily infected with VSV as the two other cancer cell lines. At this time, the cells already display a rounded and detached morphology, suggestive of cells undergoing cell death. Normal MOSE cells were more resistant to infection by VSV and JX-594. (TIFF 822 kb) [file 1750-1172-8-33-S6.tiff]
