# Supplementary material for: Small cell ovarian carcinoma: genomic stability and responsiveness to therapeutics
Source: Orphanet J Rare Dis. 2013 Feb 21;8:33. doi: 10.1186/1750-1172-8-33 (PMC3635907; doi:10.1186/1750-1172-8-33)
Supplement: Additional file 3: Table S1. — Examples of SNPs shared between BIN-67 and at least 1 tumour sample, but not the matched normal sample identified by CRMAv2 and HMMDosage analysis of the Affymetrix Genome-Wide Human SNP Array 6.0. (PDF 23 kb) [file 1750-1172-8-33-S3.pdf]

Table S1: Examples of SNPs shared between BIN-67 and at least 1 tumour sample, but not the matched normal sample identified by CRMAv2 and HMMDosage analysis of the Affymetrix Genome-Wide Human SNP Array 6.0.

Sample*	Chromosome	Start	Stop	# Probes	LogR	HMM Prediction
BIN-67	11	5744657	5765716	33	-0.6009	loss
T3	11	5746446	5765716	28	-0.4529	loss
BIN-67	10	27265910	27268480	13	0.4712	gain
T1	10	27265910	27268480	13	0.5777	gain
BIN-67	4	34462896	34501121	39	-0.7643	loss
T3	4	34462896	34501121	39	-3.4164	loss
T1	4	34462896	34501121	39	-0.7168	loss
BIN-67	2	34552819	34590018	46	-2.174	loss
T3	2	34552819	34590562	47	-2.6093	loss
BIN-67	19	56834165	56840010	11	0.7951	gain
T2	19	56834165	56840010	11	0.5216	gain
T4	19	56836959	56840010	6	0.5031	gain
BIN-67	5	57361785	57369291	32	-1.3411	loss
T2	5	57361785	57369291	32	-1.7706	loss
T3	5	57361785	57369291	32	-1.797	loss
T1	5	57361785	57369291	32	-1.543	loss
BIN-67	11	81192815	81194909	7	-0.6626	loss
T2	11	81181573	81194909	14	-0.6116	loss
BIN-67	7	109228866	109241147	10	-0.6882	loss
T4	7	109229016	109241147	9	-0.5664	loss
BIN-67	1	110046522	110047805	6	0.4223	gain
T4	1	110046522	110047805	6	0.4352	gain
BIN-67	7	142155613	142167487	42	-1.6596	loss
T1	7	142155613	142167487	42	-1.7599	loss
BIN-67	1	151028548	151035325	37	-1.953	loss
T1	1	151028548	151035325	37	-2.4155	loss
T3	1	151028548	151035325	37	-0.7248	loss
T4	1	151028548	151035325	37	-1.7965	loss
T2	1	151028671	151035325	33	-0.6367	loss
BIN-67	23	78443988	78542466	87	0.2823	gain
T2	23	78443988	78557117	95	0.3028	gain

*Samples are BIN-67 cells and SSCOHT patient tumour samples T1, T2, T3,and T4.
